# LncRNA‐mRNA competing endogenous RNA network depicts transcriptional regulation in ischaemia reperfusion injury

**DOI:** 10.1111/jcmm.14163

**Published:** 2019-01-18

**Authors:** Hongying Liu, Danping Xu, Xin Zhong, Dongsheng Xu, Geng Chen, Junbo Ge, Hua Li

**Affiliations:** ^1^ Zhongshan Hospital, Shanghai Institute of Cardiovascular Diseases, Fudan University Shanghai China; ^2^ Shanghai Green Valley Pharmaceutical Company Shanghai China; ^3^ Department of Cardiology, Guangdong Provincial Hospital of Chinese Medicine Guangzhou China; ^4^ Institutes of Biomedical Sciences Fudan University Shanghai China; ^5^ Shanghai Key Laboratory of Regulatory Biology, The Center for Bioinformatics and Computational Biology, The Institute of Biomedical Sciences and School of Life Sciences East China Normal University Shanghai China

**Keywords:** acute myocardial infarction, ceRNA, GTF2H4, lncRNA

## Abstract

The study aimed to investigate time‐course transcriptomes in myocardial ischaemia reperfusion injury (IRI) via RNA‐Seq. Transcriptomes of 10 samples derived from patients with acute ST‐segment elevation myocardial infarction (ASTEMI) who were assigned to percutaneous coronary intervention (PCI), were sequenced at the time of 0 (before PCI), 2, 12, 24 and 72 hours after PCI, respectively. Using the genefilter package in r, wgcna and stem, different expression lncRNA (DEL) and mRNA (DEM) were analysed. Out of 756 mRNAs and 206 lncRNAs shared by enrolled patients, 135 RNAs were screened to be significantly associated with the IRI. Furthermore, combined with lncRNA‐mRNA, lncRNA‐miRNA and miRNA‐mRNA network, 51 RNAs and 131 relationship pairs were ascertained in the competing endogenous RNAs (ceRNA) network. Among these nodes, SH2D3C and GTF2H4 were significantly enriched in cellular response to stress and their interaction module were isolated from functional ceRNA network. Subsequently, their critical role was confirmed via down‐regulation of SH2D3C and GTF2H4 expression in vitro model. These results identified that lncRNA‐mRNA ceRNA network, associated significantly with IRI, functioned as critical regulative pivotal roles after PCI‐AMI, and SH2D3C and GTF2H4 may be the most responsive transcriptional regulator in the early‐phase of IRI.

## 1 INTRODUCTION

Acute myocardial infarction (AMI) is the severe event of coronary artery disease.^1^ And more than seven million new patients have been diagnosed with AMI worldwide annually.^2^ Percutaneous coronary intervention (PCI) remains the primary therapeutic choice for them in recent years. However, there are still some limitations, such as ischaemia reperfusion injury (IRI). Therefore, it is very important to improve the clinic outcomes of PCI‐AMI through revealing the relative mechanisms of IRI and taking effective measures. LncRNAs in previous studies have played critical roles in the physiological and pathological processes of heart.^3^ LncRNA‐mRNA competing endogenous RNA network (ceRNA) was considered to perform a critical role in the lncRNA associated regulatory network in AMI. Furthermore, four lncRNAs (RP1‐239B22.5, AC135048.13, RP11‐4O1.2, RP11‐285F7.2) from MI‐occurrence and three lncRNAs (RP11‐363E7.4, CTA‐29F11.1, RP5‐894A10.6) from MI‐recurrence were identified as potential biomarkers through ceRNA.^4^ Hence, it is essential for physicians to deeply analyse the biological functions of ceRNA network in the perioperative period of PCI‐AMI.

## 2 MATERIALS AND METHODS

In the current study, PAXgene Blood RNA tubes (BD, San Jose, CA, USA) were used to store the peripheral blood samples of patients with acute ST‐segment elevation myocardial infarction (STEMI) at the time of 0 (before PCI), 2, 12, 24 and 72 hours after PCI respectively. Then the purified RNA was eluted and collected, according to the instructions of PAXgene Blood RNA kit (BD). This study protocol (Registration number: ChiCTR‐IPR‐17010501) was approved by the Ethics Committee of Guangdong Provincial Hospital of Chinese Medicine. All participants have been provided informed consents and all patients satisfied the inclusion and exclusion criteria. RNA integrity was assessed using the RNA Nano 6000 Assay Kit of the Bioanalyzer 2100 system (Agilent Technologies, Santa Clara, CA). About 3 μg RNA per sample was used as input material for the RNA sample preparations. The products were purified (AMPure XP system) and library quality was assessed on the Agilent Bioanalyzer 2100 system. Then, the libraries were sequenced on an Illumina Hiseq 2500 platform and 125 bp paired‐end reads were generated. The mapped reads of each sample were assembled by both Scripture (β2) (Guttman et  al, 2010) and Cufflinks (v2.1.1) (Trapnell et  al, 2010) in a reference‐based approach. Co‐efficient of variations (CVs) for samples in enrolled patients at different time‐points were calculated using genefilter (version 1.58.1, https://bioconductor.org/packages/release/bioc/html/genefilter.htmlpackage in r 3.4.1. Subsequently, different expression lncRNA (DEL) and mRNA (DEM), significantly (*P *< 0.05) changed along with the tendency of times, were screened with the threshold of CV > 0.6. Gene Ontology (GO) and the Kyoto Encyclopedia of Genes and Genomes (KEGG) functional analyses were performed for DEMs using the DAVID online tool (http://david.ncifcrf.gov/)^6^, ^7^ with the threshold of *P* < 0.05. wgcna package (version 1.61, https://cran.r-project.org/web/packages/WGCNA/)^8^ was used to analyse the associations between genes/models and IRI. Short time‐series expression miner (STEM, version 1.3.11, http://www.cs.cmu.edu/~jernst/stem/) was utilized to cluster the DELs and DEMs with significant similar expression models (*P* < 0.05) according to RNAs expression at different time‐points. The lncRNAs‐targeted mRNAs and mRNAs were combined and the lncRNA‐mRNA regulatory network was visualized using Cytoscape 3.3 (9 Finally, the miRNA‐targeted mRNA regulatory network was mapped to the lncRNA‐mRNA regulatory network for the construction of the lncRNA‐miRNA‐mRNA (ceRNA) regulatory network. The transfection of si‐GTF2H4 and si‐SH2D3C was performed according to the manufacturer's protocol of lipofectamine 3000 (Invitrogen, Carlsbad, CA, USA). The cell viability of the rat cardiac microvascular endothelial cells (CMECs) was conducted by CCK‐8 assays. (Detail methods are in Supplementary Methods)

## 3 RESULTS

After sequencing and filtering the low‐quality beads, a total of 898 681 000 clean reads were acquired (82.151% of total) (Supplementary 1A). Using the TopHat, RNAs were divided into mRNA (85.54%), lncRNA (2.12%), etc (Supplementary 1B). Pearson co‐efficient analysis showed that R^2^ of each two samples ranged from 0.731 to 0.886 (mean value = 0.837) (Supplementary 1C). Meanwhile, samples of each patient at different time‐points were prone to distribute in an identical area (Supplementary 1D). Based on the expression abundance, bidirectional hierarchical clusters of samples were demonstrated in Supplementary 1E,F. About 267 DELs and 1154 DEMs, and 405 DELs and 1887 DEMs were identified with CV >0.6 at all individual points respectively. Bidirectional hierarchical cluster of these DELs was showed in Supplementary 2A. The VENN analytical results showed that 206 DELs and 756 DEMs were shared by enrolled patients (Supplementary 2B). Following this, the 756 DEMs were subjected to the GO and KEGG functional enrichment analysis: 16 biological processes (Supplementary 2C) and 17 KEGG pathways (Supplementary 2D).

**Figure 1:**
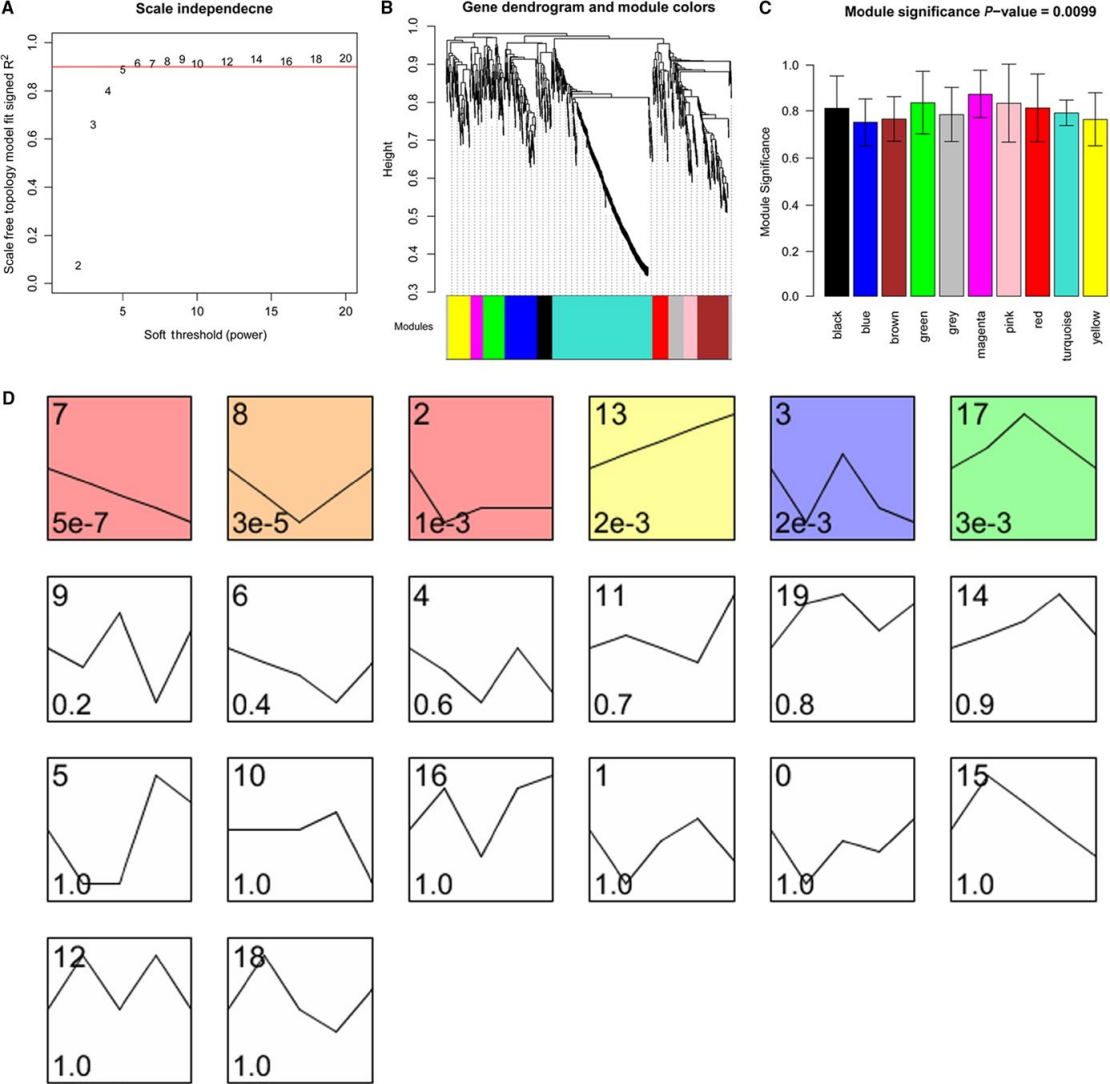
Identification of ischaemia reperfusion injury (IRI)‐associated RNAs and models. A, Result of power value selection. *X* axis represented the power value and *Y* axis represented the square of log(k) and log(p(k)). The red line represented the standard line that indicated square value = 0.9. B, Clustering of IRI‐associated RNAs. C, IRI‐associated modules. D, Clustering of RNAs varied with time tendency. Different squares indicated clusters. Number in top left corner represented the gene number, and in the left bottom represented the clustered *P* value. Table: models and RNAs associated with IRI

To obtain genes associated with IRI, 206 DELs and 756 DEMs were subjected to further analyses. When co‐efficient R^2 ^= 0.9, power = 6 was obtained and utilized for the CV calculation (Figure 1A). The cluster tree was constructed with thresholds of gene number ≥ 20 and CutHeight = 0.95, according to the criteria of hybrid dynamic shear tree (Figure 1B). Then, the co‐efficient between model and IRI was around 0.8 and the significance between whole RNAs and IRI at detected points was 0.0099 (Figure 1C, Table S1); with *P* < 0.05, 6 of time‐point significantly associated models were identified using STEM, including 48, 32, 66, 39, 44 and 54 RNAs respectively (Figure 1D).

**Figure 2:**
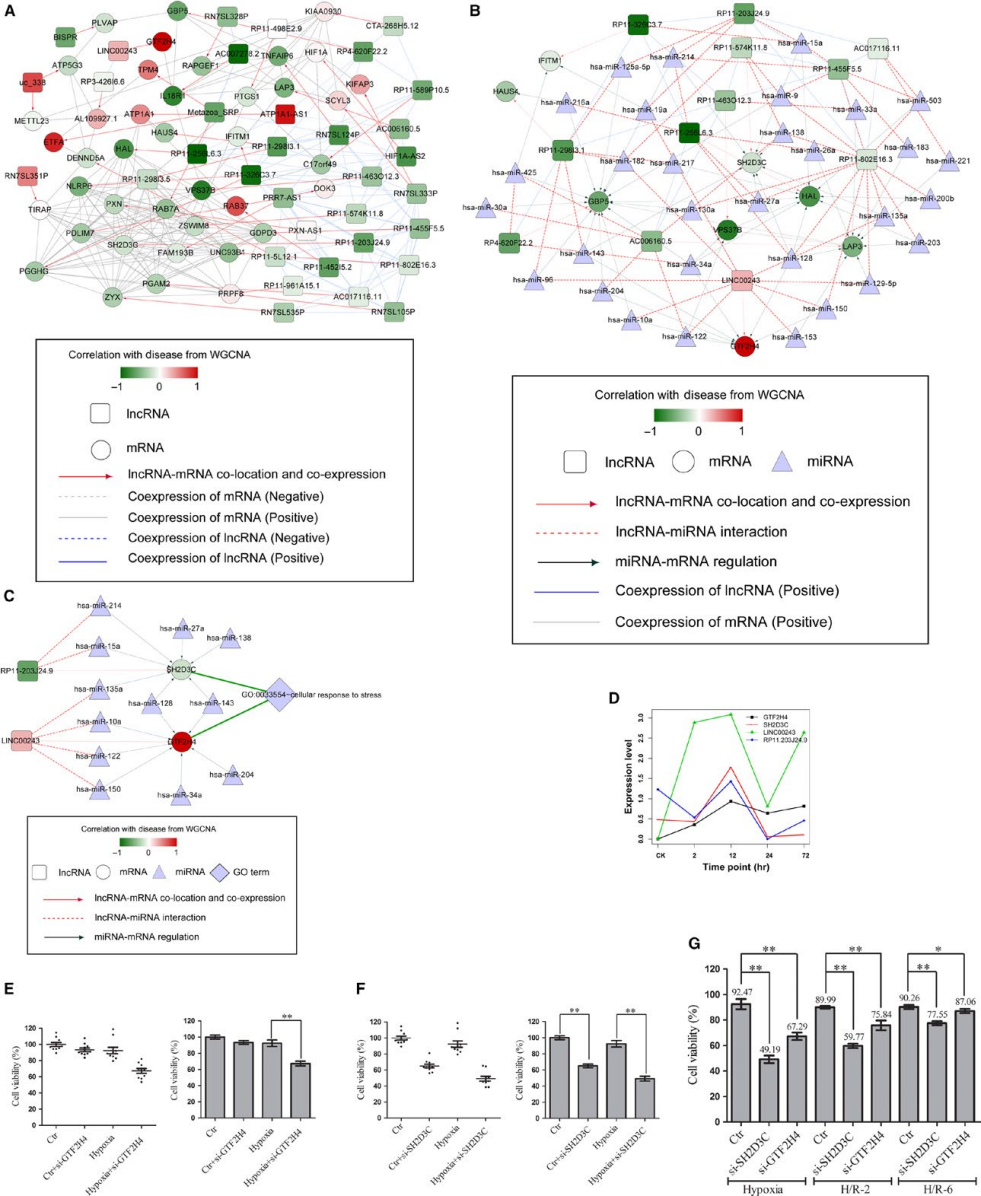
Regulatory network analysis. A, LncRNA‐mRNA regulatory network. B, LncRNA‐miRNA‐mRNA (ceRNA) regulatory network. C, SH2D3C‐ and GTF2H4‐associated ceRNA network. D, Expression of SH2D3C, GTF2H4, RP11‐203J24.9 and LINC00243 at different time‐points. E, Cardiac microvascular endothelial cell (CMEC) viability was conducted by CCK‐8 assays between control group and si‐GTF2H4 group. The viability of si‐GTF2H4 group was significantly decreased than the control group under hypoxia condition (*P *< 0.05). F, CMEC viability was conducted by CCK‐8 assays between control group and si‐SH2D3C group. The viability of si‐SH2D3C group was significantly decreased than the control group under both normal and hypoxia condition (*P *< 0.05). G, CMEC viability was conducted by CCK‐8 assays among control group, si‐SH2D3C group and si‐GTF2H4 group at given hypoxia‐reoxygenation time‐points: Hypoxia (H/R‐0 h), H/R‐2 h, H/R‐6 h. The viabilities of si‐SH2D3C group and si‐GTF2H4 group were both significantly decreased than the control group under at three different time‐points (*P *< 0.05)

About 18 997 lncRNA‐mRNA pairs and 105 188 RNA expression associated pairs were combined to select the overlapped lncRNA‐mRNA pairs; 42 pairs were selected to construct the lncRNA‐mRNA regulatory network (Figure 2A). According to miRcode and starBase databases, the lncRNA‐miRNA and miRNA‐mRNA regulatory networks were combined to construct the ceRNA regulatory network (Figure 2B). Furthermore, functional enrichment analyses of mRNA involved in ceRNA network were deeply analysed. Only SH2D3C and GTF2H4 were found to significantly (*P* < 0.05) enrich in GO_BP: GO:0033554 ~ cellular response to stress (Figure 2C). The module contained 16 nodes: two lncRNAs, two mRNAs, 12 miRNAs, and formed 22 edges: two lncRNA‐mRNA pairs, six lncRNA‐miRNA pairs and 14 miRNA‐mRNA pairs. The peak was found at 12 hours after PCI (Figure 2D). Especially, both SH2D3C and RP11‐203J24.9 presented negative correlations with IRI and could be targeted by hsa‐miR‐15a and hsa‐miR‐214; GTF2H4 and LINC00243 presented positive correlations with IRI and could be regulated by hsa‐miR‐10a, hsa‐miR‐122 and hsa‐miR‐150. To demonstrate the critical roles of SH2D3C and GTF2H4 in IRI, we did experiments in in vitro model (hypoxia/reoxygenation injury) to provide further evidence by down‐regulating SH2D3C and GTF2H4 expression. The results showed that the viability of cardiac microvascular endothelial cells was significantly reduced in the process of hypoxia/reoxygenation injury and in the presence of si‐SH2D3C and si‐GTF2H4 (Figure 2E‐G).

## 4 CONCLUSION

Our results identified that lncRNA‐mRNA ceRNA network, associated significantly with IRI, functioned as critical regulative pivotal roles after PCI‐AMI and SH2D3C and GTF2H4 could be the most responsive transcriptional regulators in the early‐phase of IRI.

## CONFLICTS OF INTERESTS

The authors declare that there is no conflict of interests regarding the publication of this paper.
