# Supplementary material for: LncRNA‐mRNA competing endogenous RNA network depicts transcriptional regulation in ischaemia reperfusion injury
Source: J Cell Mol Med. 2019 Jan 18;23(3):2272–6. doi: 10.1111/jcmm.14163 (PMC6378211; doi:10.1111/jcmm.14163)
Supplement: Supplementary file 1 [file JCMM-23-2272-s001.docx]

**LncRNA-mRNA competing endogenous RNA network depicts transcriptional regulation in ischemia reperfusion injury**

Hongying Liu^1,2*^, Danping Xu^3*^, Xin Zhong^1^, Dongsheng Xu^4^, Geng Chen^5^, Hua Li^1#^

^1^Shanghai Institute of Cardiovascular Diseases, Zhongshan Hospital, Fudan University, Shanghai 200032, China

^2^Shanghai Green Valley Pharmaceutical Company, Shanghai 201203, China

^3^Department of Cardiology, Guangdong Provincial Hospital of Chinese Medicine, Guangzhou 510120, China

^4^Institutes of Biomedical Sciences, Fudan University, Shanghai 200032, China

^5^The Center for Bioinformatics and Computational Biology, Shanghai Key Laboratory of Regulatory Biology, the Institute of Biomedical Sciences and School of Life Sciences, East China Normal University, Shanghai 200241, China

^*^ Hongying Liu and Danping Xu contributed equally to this work.

^#^Correspondence to: Hua Li, M.D. & Ph.D.

Shanghai Institute of Cardiovascular Diseases, Zhongshan Hospital, 180 Fenglin Road, Fudan University, Shanghai, China, 200032

Tel.: +86 21 60268574

Fax: +86 21 60268574

E-mail: [lihua199988@hotmail.com](mailto:lihua199988@hotmail.com)

**Keywords**: acute myocardial infarction; lncRNA; ceRNA; GTF2H4; SH2D3C

**Suppl Methods**

**Patients and sampling**

Patients in this study were enrolled if they met the following criteria: 1) aged between 55 and 75 years, 2) presented ≤12 hours after the onset of symptoms and with direct percutaneous coronary intervention (PCI) and depside salt from salvia miltiorrhiza (1/day, 0-7^th^ day, Green Valley lnc., Shanghai, China), and 3) received 300 mg of aspirin and a loading dose of adenosine diphosphate receptor antagonists (300–600 mg clopidogrel) in the emergency room. Patients were excluded if they were met any of the following term: 1) fibrinolysis, 2) left bundle-branch block in the presenting electrocardiogram, 3) cardiogenic shock, and 4) bypass surgery within the previous month.

[PAXgene Blood RNA t](https://www.baidu.com/link?url=1Sg8TDtBY_s9jxi40PkKAO99iuolygUGbYBdWkyhFYO8fXnK21gdl-qClndUBS9DCEWD4W_GivSCFuxPXyZ8L6FsrB-mTUGikEZuiD1_tIe&wd=&eqid=8a5c10850000a3e70000000359e99fe0)ubes (BD, USA) were used to store the peripheral blood samples of patients with AMI at the time of 0 (before PCI), 2, 12, 24, and 72h after PCI，respectively. Then the purified RNA was eluted and collected, according to the instructions of PAXgene Blood RNA kit (BD, USA). This study protocol (Registration number: ChiCTR-IPR-17010501) was approved by the Ethics Committee of Guangdong Provincial Hospital of Chinese Medicine. All participants have been provided informed consents and all patients satisfied the inclusion and exclusion criteria.

**RNA isolation, RNA quantification and qualification**

Total RNA from human blood was isolated with TRIzol reagent (Invitrogen, Carlsbad, CA) according to the manufacturer’s protocol. RNA degradation and contamination were monitored on 1% agarose gels. RNA purity was checked using the NanoPhotometer spectrophotometer (IMPLEN, CA, USA). RNA concentration was measured using Qubit RNA Assay Kit in Qubit 2.0 Flurometer (Life Technologies, CA, USA). RNA integrity was assessed using the RNA Nano 6000 Assay Kit of the Bioanalyzer 2100 system (Agilent Technologies, CA, USA).

**Construction of lncRNA library**

A total amount of 3 μg RNA per sample was used as input material for the RNA sample preparations. 1) Ribosomal RNA was removed by Epicentre Ribo-zero rRNA Removal Kit (Epicentre, USA), and rRNA free residue was cleaned up by ethanol precipitation. 2) Sequencing libraries were generated using the rRNA-depleted RNA by NEBNext Ultra Directional RNA Library Prep Kit for Illumina (NEB, USA) following manufacturer’s recommendation. Briefly, fragmentation was carried out using divalent cations under elevated temperature in NEBNext First Strand Synthesis Reaction Buffer (5X). First strand cDNA was synthesized using random hexamer primer and M-MuLV Reverse Transcriptase (RNaseH-). Second strand cDNA was subsequently synthesized using DNA Polymerase I and RNase H. In the reaction buffer, dNTPs with dTTP were replaced by dUTP. Remaining overhangs were converted into blunt ends via exonuclease/polymerase activities. After adenylation of 3’ ends of DNA fragments, NEBNext Adaptor with hairpin loop structure were ligated to prepare for hybridization. In order to select cDNA fragments of preferentially 150~200 bp in length, the library fragments were purified with AMPure XP system (Beckman Coulter, Beverly, USA). 3) 3 μl USER Enzyme (NEB，USA) was used with size-selected, adaptor-ligated cDNA at 37°C for 15 min followed by 5 min at 95°C before PCR. 4) PCR was performed with Phusion High-Fidelity DNA polymerase, Universal PCR primers and Index (X) Primer with 12 cycles. 5) Products were purified (AMPure XP system) and library quality was assessed on the Agilent Bioanalyzer 2100 system.

**Clustering and sequencing**

The clustering of the index-coded samples was performed on a cBot Cluster Generation System using TruSeq PE Cluster Kit v3-cBot-HS (Illumia) according to the manufacturer’s instruction. After cluster generation, the libraries were sequenced on an Illumina Hiseq 2500 platform and 125 bp paired-end reads were generated.

**Quality control**

Raw data (raw reads) of fastq format were first processed through in-house perl scripts. During this step, clean data (clean reads) were obtained by removing reads containing adapter, reads on containing ploy-N and low-quality reads from raw data. At the same time, Q20, Q30 and GC content of the clean data were calculated. All the downstream analyses were based on the clean data with high quality.

**Mapping to the reference genome**

Reference genome and gene model annotation files were downloaded from genome website directly. Index of the reference genome was built using Bowtie v2.0.6 and paired-end clean reads were aligned to the reference genome using TopHat v2.0.9.

**Transcriptome assembly**

The mapped reads of each sample were assembled by both Scripture (beta2) (Guttman et al. 2010) and Cufflinks (v2.1.1) (Trapnell et al. 2010) in a reference-based approach. Spliced reads were used to determine exons connectivity in both methods, but with two different approaches. Scripture employed a statistical segmentation model to distinguish expressed loci from experimental noise and used spliced reads to assemble expressed segments. It would indicate all statistically expressed isoforms in a given locus. Cufflinks employed a probabilistic model to simultaneously assemble and quantify the expression level of a minimal set of isoforms that provided a maximum likelihood explanation of the expression data in a given locus (Cabili et al. 2011). Scripture was run with default parameters, Cufflinks was run with ‘min-frags-per-transfrag=0’ and ‘--library-type’, other parameters were set as default.

**Pre-progressing of raw data**

To obtain relative high-quality data (clean reads), adaptor sequences and beads with low quality were removed out from the raw beads using using FastQC (<http://www.bioinformatics.babraham.ac.uk/projects/fastqc/>). Then, clean reads were mapped to human genome Release 27 (GRCh38. p10, [https: //www.gencodegenes.org /releases/current.html](https://www.gencodegenes.org/releases/current.html)) provided by GENCODE and annotated via tophat (version 2.1.1, [http: //ccb.jhu.edu/software/tophat/index.shtml](http://ccb.jhu.edu/software/tophat/index.shtml)) ^12^. After that, expression abundance at different time points were calculated using cufflinks (version 2.2.1, [http: //cole-trapnell-lab.github.io/cufflinks/](http://cole-trapnell-lab.github.io/cufflinks/)) ^13^ and shows with FPKM (Reads Per Kilobase of exon model per Million mapped reads).

**Comparison and analysis of expression abundance**

Based on the expression abundance, several methods were applied to test the correlation between samples. 1) Pearson co-efficient between each two samples was calculated using COR function ([https: //stat.ethz.ch/R-manual /R-devel/library/stats/html/cor.html](https://stat.ethz.ch/R-manual/R-devel/library/stats/html/cor.html)) in R 3.4.1 and presented with R^2^.The more close to 1 the R^2^ was, the more similarity between samples presented. 2) the expression abundance cluster of samples was conducted using the correlation method in pheatmap (version 1.0.8, [https: //cran.r-project.org/web /packages/pheatmap/index.html](https://cran.r-project.org/web/packages/pheatmap/index.html)) ^14^ in R 3.4.1. 3) principal components analysis (PCA) of samples was performed based on the expression abundance using the psych package (version 1.7.8, [https: //cran.r-project.org/web /packages/psych/index.html](https://cran.r-project.org/web/packages/psych/index.html)).

**Identifications of differentially expressed lncRNAs (DELs) and mRNAs (DEMs)**

Co-efficient of variations (CVs) for samples in enrolled patients at different time points were calculated using genefilter (version 1.58.1, [https: //bioconductor.org /packages/release/bioc/html/genefilter.html](https://bioconductor.org/packages/release/bioc/html/genefilter.html)) ^15^ package in R 3.4.1. Then, DELs and DEMs, which were significantly changed along with the tendency of times, were screened with the threshold of CV>0.6. Finally, Gene Ontology (GO) and the Kyoto Encyclopedia of Genes and Genomes (KEGG) functional analyses were performed for DEMs using the DAVID online tool (http://david.ncifcrf.gov/) ^16,17^ with the threshold of P <0.05.

**Selection of IRI associated DELs and DEMs**

WGCNA package (version 1.61, https://cran.r-project.org /web/packages/WGCNA/) ^18^ was used to analyze the associations between genes/models and IRI. Simultaneously, short time-series expression miner (STEM, version 1.3.11, <http://www.cs.cmu.edu/~jernst/stem/>) was utilized to cluster the DELs and DEMs with significant similar expression models (P <0.05) according to RNAs expression at different time points. In the end, combined results of WGCNA and STEM, IRI associated DELs and DELs with significant expression changes and similar expression tendency, were selected from DEGs isolated with CVs and utilized for the further analysis.

**Construction of lncRNA-mRNA regulatory network**

The lncRNA-mRNA network was built to further reveal the biofunction of lncRNA. RNA locations were first acquired, based on the previous mapping information. Meanwhile, according to locations of lncRNAs, lncRNAs targeted mRNAs were identified from 1000 kb of the up- and down-stream of their location in the chromosome. Following this, based on the coefficient obtained by WGCNA, RNAs, which disease associated coefficient >0.6, were selected to construct the network. Combined the lncRNAs-targeted mRNAs and mRNAs, the lncRNA-mRNA regulatory network was visualized using Cytoscape 3.3 ([http: //www.cytoscape.org/](http://www.cytoscape.org/)) ^19^.

**Construction of ceRNA regulatory network**

Based on the miRecode (version 11, <http://www.mircode.org/>) and starBase (version 2.0, <http://starbase.sysu.edu.cn/index.php>), the overlapped regulatory relationships between lncRNAs and miRNAs were screened to construct lncRNA-miRNA regulatory network. Then, miRNA-target regulatory relationships were identified from miRTaeBase (version 6.0, <http://mirtarbase.mbc.nctu.edu.tw>), which provides experimental validated miRNA-target regulatory results. Subsequently, the miRNA-targeted mRNA regulatory network was mapping to the lncRNA-mRNA regulatory network. And the lncRNA-miRNA-mRNA (ceRNA) regulatory network was constructed. In addition, functional analyses of mRNAs involved in the ceRNA network were performed.

**siRNA transfection**

The rat CMECs were grown to 50% confluency on 6-well plates, the transfection was produced by the manufacturer's protocol of lipofectamine 3000 (invitrogen, USA). Briefly, 1.5µL Gtf2h4/Sh2d3c-siRNA or Negative Control-RNA and 3µL lipofectamine 3000 were diluted in 100µL serum-free DMEM medium, mixed it fully and incubated for 20min at RT. Then, the mixture was added into the 6-well plates and cultured at 37℃ for 36 hours. After 36 hours, the cells were harvested. The expression of Gtf2h4 or Sh2d3c expression were detected by qPCR.

si-GTF2H4: GGUACUUCAUGCUGCAGUATT；

si-SH2D3C: GGUUCCACACCAUGUCCAUTT.

**Total RNA extraction and qRT-PCR analysis**

Total RNA was extracted by the FastPure Cell/Tissue Total RNA Isolation Kit (Vazyme, China) and stored at -80℃. The cDNA was synthesised according to the manufacturer's instructions of the HiScript II Q RT SuperMix for qPCR (Vazyme, China). qRT-PCR analysis was performed as described using the Maxima SYBR Green/ROX qPCR Master Mix (Thermo, USA) in CFX Connect Real-Time System (Bio-Rad Laboratories, USA), with β-actin considered as the internal control. The relative expression levels of the target genes were calculated according the 2^-ΔΔCt^ method. All sample measurements were conducted in triplicate. The primers were synthesised by TsingKe Biological Technology (Beijing, China), and the primers sequence of qRT-PCR were as follows:

Gtf2h4:

forwad: CCAGCAGATTATCCATTTCCTAA,

reverse: CAGCAGCAATTCAAAGTCCA;

Sh2d3c:

forward: GCCACCTCATCTTTCAACC,

reverse: CGCCCAGTATCCTAGCAACC;

β-actin:

forward: GTAAAGACCTCTATGCCAACA,

reverse: GGACTCATCGTACTCCTGCT.

**Cell viability assay**

Cell viability was conducted by CCK-8 assays. The rat cardiac microvascular endothelial cells (CMECs) were seeded into 96-well plates at a density of 1*10^4^ cells per well. Overnight incubation, the cells were cultured in a three-gas hypoxic incubator chamber (5% CO_2_, 1% O_2_, 94% N_2_) for 4 hours, and then cultured in normal incubator chamber at given time points: Hypoxia(H/R-0h), H/R-2h, H/R-6h. After that, according to the manufacturer's instructions, the 10µL CCK-8 reagent (Beyotime Biotechnology, China) was added into each well and incubated for 1 hours at 37℃ for further measurement. The absorbance was detected at 450 nm.

**Statistical significance test**

Differentially expressed lncRNAs (DELs) and mRNAs (DEMs) were identified by R package of genefilter (version 1.58.1, [https: //bioconductor.org /packages/release/bioc/html/genefilter.html](https://bioconductor.org/packages/release/bioc/html/genefilter.html))^15^, and adjusted p-values < 0.05 calculated using the method of Benjamini and Hochberg was used as the significance cutoff. Similar expression models were analyzed according to RNAs expression at different time points by employing STEM (short time-series expression miner, version 1.3.11, <http://www.cs.cmu.edu/~jernst/stem/>), those significant models were defined as the ones with p-value < 0.05 calculated by STEM. The significance of co-efficient between model and IRI (ischemia reperfusion injury) was calculated by WGCNA package (version 1.61, https://cran.r-project.org /web/packages/WGCNA/) ^18^, and p-value < 0.05 was considered as significant. Functional enrichment analyses of GO terms and pathways were conducted using the online tool of DAVID (The Database for Annotation, Visualization and Integrated Discovery, <https://david.ncifcrf.gov/>), and the method of Benjamini and Hochberg was employed to get adjusted p-values, where 0.05 was defined as the significance threshold.

**Suppl. Table**

**Suppl Table 1. Models and RNAs Associated with IRI**

| **Module Color** | **Correlation with Disease** | **Gene number** | **lncRNA** | **mRNA** |
| --- | --- | --- | --- | --- |
| black | 0.8254 | 51 | 11 | 40 |
| blue | 0.7806 | 109 | 24 | 85 |
| brown | 0.7898 | 105 | 16 | 89 |
| green | 0.8349 | 74 | 15 | 59 |
| grey | 0.7945 | 68 | 20 | 48 |
| magenta | 0.8494 | 42 | 10 | 32 |
| pink | 0.834 | 46 | 13 | 33 |
| red | 0.8259 | 54 | 9 | 45 |
| turquoise | 0.8173 | 338 | 76 | 262 |
| yellow | 0.7876 | 75 | 12 | 63 |

**Suppl. Figures & Legends**

**Supplementary Figure 1:**


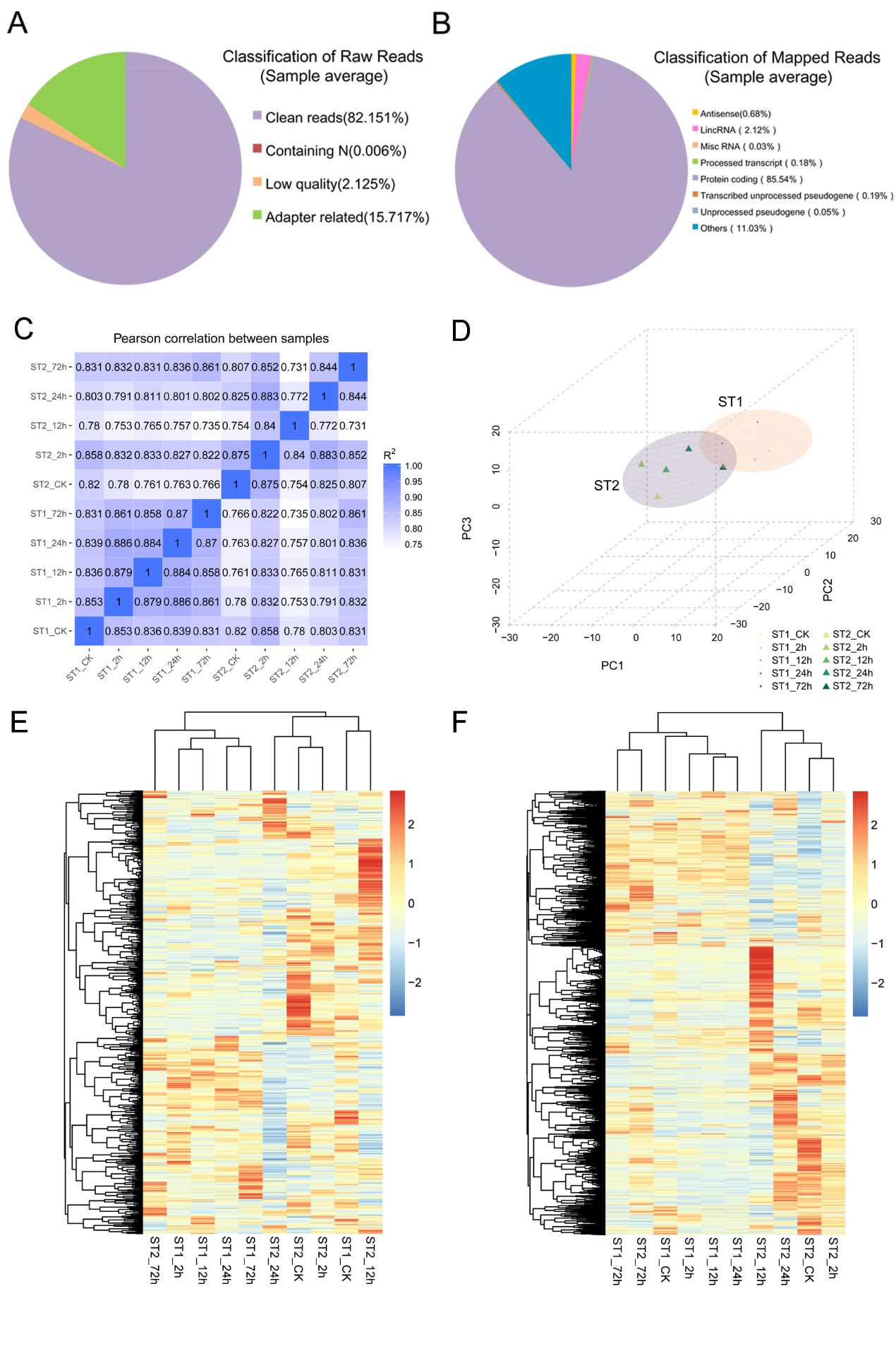


**Suppl. Fig.1 Transcriptome sequencing results and PCA analyses of RNAs based on the expression levels.**

A, Pie chart for the raw sequencing data; B, Pie chart for raw data after mapping to human reference genome. C, association between each two samples. Number in square represented the co-efficient R^2^ and X/Y axis represented the sample. Color of square was positive correlated with the size of R^2^. D, PCA analysis for RNAs. X, Y, and Z axes represented the primary component 1, 2, and 3, respectively. Red and green dots represented the samples of ST1 and ST2 patients at different time points. E&F, Bidirectional hierarchical cluster heatmap for lncRNA (left) and mRNAs (right).

**Supplementary Figure 2:**


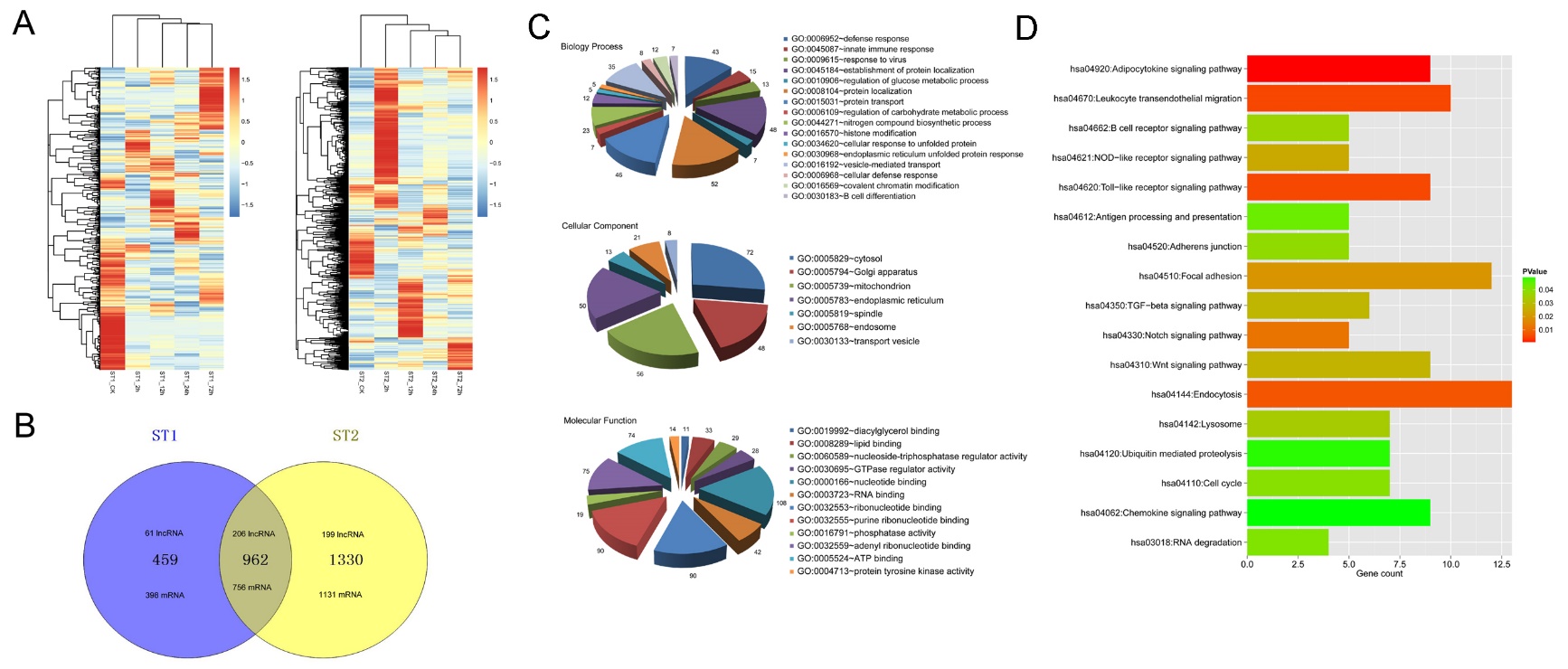


**Suppl. Fig.2 DELs and DEMs identified in enrolled patients and GO and KEGG functional enrichment analyses.**

A, Heatmaps for DELs and DEMs in ST1 (left) and ST2 (right) patients; B, Venn diagram analysis for DELs and DEMs identified in ST1 and ST2 patients. C, Pie chart for GO functional enrichment analysis. Different color represented different GO terms and number represented gene numbers enriched in this term. D, KEGG analysis for shared DELs and DEMs. X axis represented the mRNA number enriched in this pathway and Y axis represented the KEGG pathways. GO, Gene Ontology; KEGG, Kyoto Encyclopedia of Genes and Genomes; DEL, differentially expressed long non-coding RNA; DEM, differentially expressed mRNA.
